# Progress in the Use of Antisense Oligonucleotides for Vaccine Improvement

**DOI:** 10.3390/biom10020316

**Published:** 2020-02-17

**Authors:** Alexander Batista-Duharte, Luis Sendra, Maria José Herrero, Damiana Téllez-Martínez, Iracilda Zeppone Carlos, Salvador Francisco Aliño

**Affiliations:** 1 School of Pharmaceutical Sciences, Department of Clinical Analysis, São Paulo State University (UNESP), Rod. Araraquara-Jaú - Km 1, 14800-903 Araraquara, SP, Brazil; damianatellezm@gmail.com (D.T.-M.); carlosiz@fcfar.unesp.br (I.Z.C.); 2 Pharmacology Department, Faculty of Medicine, Universidad Valencia, Av. Blasco Ibáñez 15, 46010 Valencia, Spain; luis.sendra@uv.es (L.S.); salvador.f.alino@uv.es (S.F.A.)

**Keywords:** antisense oligonucleotide, vaccines, adjuvants, infectious disease, cancer

## Abstract

Antisense oligonucleotides (ASOs) are synthetically prepared short single-stranded deoxynucleotide sequences that have been validated as therapeutic agents and as a valuable tool in molecular driving biology. ASOs can block the expression of specific target genes via complementary hybridization to mRNA. Due to their high specificity and well-known mechanism of action, there has been a growing interest in using them for improving vaccine efficacy. Several studies have shown that ASOs can improve the efficacy of vaccines either by inducing antigen modification such as enhanced expression of immunogenic molecules or by targeting certain components of the host immune system to achieve the desired immune response. However, despite their extended use, some problems such as insufficient stability and low cellular delivery have not been sufficiently resolved to achieve effective and safe ASO-based vaccines. In this review, we analyze the molecular bases and the research that has been conducted to demonstrate the potential use of ASOs in vaccines.

## 1. Introduction

For more than two centuries, vaccines have contributed to the eradication or control of important diseases, and they have participated greatly in the increased life expectancy and the improvement of sanitary conditions throughout the world [1]. However, despite the great success of vaccination in public health, there are still many challenges. The lack of effective vaccines against several diseases such as HIV/AIDS, malaria, and leishmaniasis; the re-emergence of other diseases such as tuberculosis; and the appearance of new pathogenic organisms or known pathogens with increased virulence stimulate the search for more effective vaccines than those available today [2,3].

The last decades have been marked by important advances in vaccine research and development. The technology of recombinant DNA and the synthesis of peptides, the development of modern bioinformatic tools, and the use of improved adjuvants and delivery systems have allowed the development of more effective and safer vaccines based on rational designs [2,4]. Moreover, the successful completion of the human genome project in the early 2000s ushered the genomics revolution, which is beginning to have a great impact on vaccine research [4,5].

For many years, nucleic acids and short nucleotide molecules have been used as vaccine components. Their use has ranged from DNA [6,7] or RNA vaccines [8,9], to oligonucleotide sequences containing unmethylated cytidine phosphate guanosine (CpG) motifs with significant immunostimulatory (adjuvant) properties [10,11]. More recently, strategies to manipulate the expression of genes controlling the immune response or the expression of antigens of interest are being used to improve immunogenicity and vaccine efficacy.

Antisense oligonucleotides (ASOs) are synthetically prepared short single strands (usually 18–21 deoxynucleotides in length), complementary to a preRNA or an mRNA sequence of the target gene. ASOs modify the expression of specific target genes, by either splicing modifications or by recruiting RNase H leading to RNA degradation of RNA–DNA hetero-duplex, thus blocking the expression of the target gene [12,13]. The availability of human genome sequence information, freely and publicly, offers the possibility to obtain inexpensive specific synthetic oligonucleotides designed against a specific target gene. The strengths of their pharmacological effects evidenced in in vitro and in vivo models have favored the development of several drugs based on oligonucleotides that have been approved by the FDA [14]. Currently, several groups are making efforts to improve vaccine efficacy using ASOs with encouraging advances achieved over the last years, but some problems are still hampering further progress on these fronts. These are mainly related to ASOs bioavailability and the occurrence of potential off-target effects. In this review, we focus on the recent design of ASOs that have yielded promising results in terms of vaccine immunogenicity improvement. Current challenges and opportunities are also analyzed.

### 1.1. Earlier Uses of Oligonucleotides in Vaccines

In 1893, William Coley reported that a mixture of bacterial cell lysate, named Coley’s toxin, could reduce the progression of some carcinomas [15]. Since the first description of the possible anti-tumor effect of Coley’s toxin, there was much debate about its mechanism of action. More than 60 years after Coley’s report, Taliaferro and Jaroslow [16] reported that preparations of nucleases-degraded DNA and RNA could partially restore hemolysin production after a single intravenous injection of sheep red blood cells (RBC) in rabbits that received 400 r total body X radiation. However, the development of synthetic oligonucleotides for medical use was only possible after the discovery of two chemical modifications, namely 2’fluoro (2´-F) substitutions [17,18], and Phosphorothioate (PTO) chemistry [19]. Another important 2´ modification was developed in 1969, 2´-O-Methyl (2´-O-Me) [20], a major alternative in many synthetic oligonucleotides. These chemical modifications improved the cellular uptake of oligonucleotides and conferred protection against enzymatic degradation.

Several studies showed that oligonucleotides can stimulate the production of specific antibodies in mature animals after concurrent administration of an antigen with either DNA or RNA digest [21,22]. At the same time, the immunogenic capacity of nucleic acids and their influence in autoimmune diseases was demonstrated [23]. Moreover, Field et al. identified that complexes of polyinosinic and polycytidylic acids (poly (I:C)) were highly active as inducers of interferon [24]. The biological basis for this observation was understood more than three decades later when Toll-like receptor 3 (TLR3) was reported to be the receptor for double-stranded RNA [25]. Related to these findings, Tokunaga et al. identified bacterial DNA as the underlying component of a fraction extracted from *Mycobacterium bovis* strain BCG that elicited an antitumor response in different in vitro and in vivo models [26]. After that, these researchers cloned mycobacterial genes, synthesized diverse oligodeoxynucleotides (ODNs), and observed that certain palindromes in these ODNs were responsible for activating the immune response [27,28].

In 1995, Krieg et al. reported that unmethylated CpG dinucleotides (CpG ODN) within bacterial DNA activate host defense mechanisms leading to innate and adaptive immune responses [29]. CpG ODN is a ligand of Toll-like receptor 9 (TLR-9) in antigen-presenting cells (APCs). CpG ODN/TLR-9 interaction induces an innate immune response that promotes the subsequent development of adaptive immunity [10]. CpG ODN can be divided into classes A, B, C, P, and S [30]. Their utility as vaccine adjuvants has been evaluated in different clinical trials and the achieved results indicate that CpG ODN augments the induction of vaccine-specific cellular and humoral responses [11]. In 2017, the FDA approved HEPLISAV-B, the first vaccine with a CpG ODN as an adjuvant for hepatitis B vaccines [31]. On the other hand, it has been reported that CpG ODN can induce high levels of pro-inflammatory cytokines, with potential risk for developing or worsening autoimmune diseases and systemic inflammatory response syndrome (SIRS) [32,33,34,35].

### 1.2. Birth of ASOs

In 1978, Zamecnik and Stephenson used a synthetic ASO, which was complementary to 13 nucleotides of Rous sarcoma virus (RSV) RNA, to inhibit the translation of the viral RNA and subsequently block the virus replication in a chick embryo fibroblasts culture [36,37]. One year later, Donis-Keller reported that RNase H catalyzes the cleavage of the RNA strand in RNA/DNA heteroduplexes [38] in a site-specific manner. That report demonstrated for the first time that ASOs can work through an enzyme-mediated process in addition to steric blocking. The decade of the 80s was marked by other advances. In 1983, Simons and Kleckner showed evidence of the existence of naturally occurring antisense RNAs and suggested a role in the regulation of gene expression [39]. After that report, other authors successfully inhibited mRNA translation by anti-sense RNA [40,41,42,43]. Moreover, in that decade, different methods for the automatic synthesis of oligonucleotides were developed [44,45] and the first antisense patent was presented in 1987, although this was publicly available from 1995 [46].

Despite the advances achieved, the experimental and clinical use of unmodified ASOs was limited as they were easily degraded by intracellular endonucleases and exonucleases, usually via 3′-5′ activity [47]. Thus, diverse chemical modifications have been developed to protect them against nuclease degradation, increase their affinity and potency, extend their tissue half-life, and reduce the undesired off-target effects (Table 1).

After these first reports, notable progress has been made in ASOs pharmacology and now different therapeutic ASOs against different diseases, including neurodegenerative, cardiovascular, metabolic, inflammatory, infectious, and neoplastic diseases are being tested in clinical trials, and several ASOs have been approved by the US Food and Drug Administration (FDA) to be used in humans. Fomivirsen (Vitravene) was the first antisense drug approved in 1998 for cytomegalovirus (CMV) retinitis [48]. In January 2013, the FDA approved mipomersen (Kynamro, Genzyme) for homozygous familial hypercholesterolemia (HoFH) [49]. Both fomiversen and mipomersen were developed by Isis Pharmaceuticals. More recently, nusinersen was approved for spinal muscular atrophy [50] and eteplirsen for Duchenne Muscular Dystrophy [53]. There are many other ASOs in different phases of clinical trials [14,54,55,56].

## 2. Molecular Basis of ASOs

ASOs and other forms of genetic therapies have emerged as an attractive therapeutic alternative to monoclonal antibodies. Factors such as structure, stability, cellular, bioavailability, lack of immunogenicity, specificity, relative low toxicity, and low cost indicate a great future perspective for these molecules (Table 2).

### 2.1. Pharmacokinetics

Different factors such as the type of chemical modifications, the electric charge, and the use of delivery systems are pivotal factors in the pharmacokinetic properties of ASOs, independently of nucleotide sequence [57,58,59]. Following intravenous or subcutaneous administration, ASOs quickly pass from the injection site to the circulation, and the highest plasma concentrations for first and second-generation of ASOs are reached within 3–4 h with a rapid distribution phase to tissues in minutes, followed by a slow elimination from tissues lasting a few hours [58]. Double-stranded RNA ASOs and single-stranded neutral-backbone ASOs such as morpholinos exhibit low serum protein binding, with limited tissue distribution, and they are rapidly eliminated by renal filtration [58,60]. In contrast, first-generation PTO can interact with albumin and other serum proteins, extending their circulation from minutes to a few hours and their tissue distribution [61]. Studies suggest that PTO-modified ASOs can also interact with a large number of proteins on the cell surface and in the extracellular matrix and can be endocytosed into intracellular vesicles [62,63,64] After that, they bind to proteins that transport them into the nucleus and perhaps promote the hybridization to RNAs [64,65,66]. The clearance and elimination of PTO are facilitated by enzymatic degradation mediated by endo- and exonucleases that results in small-molecular-weight fragments that are easily eliminated in urine [58]. Liver, kidneys, bone marrow, adipocytes, and lymph nodes are the major systemic tissues of distribution [58,62] Second-generation ASOs with 2’-O-alkyl modifications of the ribose are metabolized more slowly than the first and third generation of ASOs, and the clearance half-lives occur in 2–4 weeks [58].

### 2.2. Mechanism of Action of ASOs

**Figure 1:**
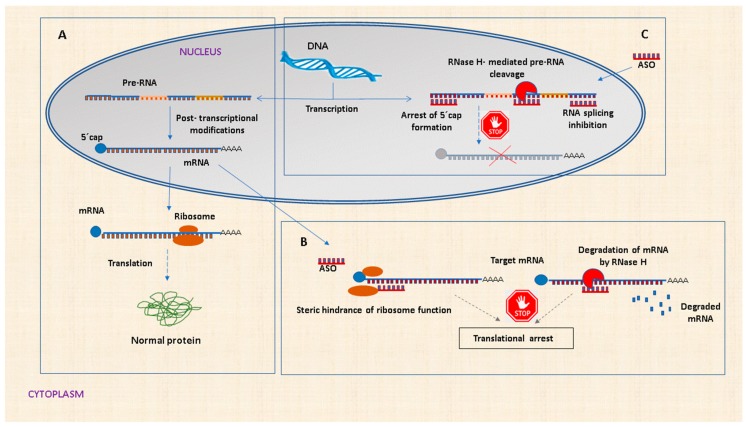
Main mechanisms of action of antisense oligonucleotides. (**A**) Normal gene and protein expression in the absence of ASO. (**B**) In cytoplasm, ASOs can bind to a complementary mRNA region. ASO-mRNA heteroduplex can induce the activation of RNase H, leading to mRNA degradation. Alternatively, ASOs can block the translation process without promoting RNA degradation by steric interference of ribosomal assembly. (**C**) ASO can enter the nucleus and hinder mRNA maturation by inhibition of 5′ cap formation, RNase H-mediated pre-RNA cleavage, and inhibition of mRNA splicing.

ASOs are designed to specifically block the transfer of the genetic information for the protein synthesis by binding to a target RNA through Watson–Crick base pairing: A-T, C-G interaction [47]. They can modulate the processing, stability, or activity of specific RNAs by different mechanisms that have been extensively analyzed in previous reviews [56,67,68]. Briefly, ASOs can directly stick to pre-RNA or mRNA molecules and prevent the formation of the 5’-mRNA cap, modulate alternative splicing, dictate the location of the polyadenylation site, and recruit RNAse H to cleave and degrade the RNA target in the ASO-RNA complex. ASOs can also sterically block the ribosomal subunits from attaching or running along with the mRNA transcript, hampering the translation process (Figure 1).

### 2.3. Toxicology of ASOs

In addition to the Watson–Crick interaction, ASOs can form other interactions, such as electrostatic links with polycations and positively charged proteins, noncanonical base-pairing with themselves and other nucleic acids, and sequence-specific interaction with proteins [69]. These non-antisense interactions can cause different toxicity manifestations [70,71,72,73,74,75].

**Figure 2:**
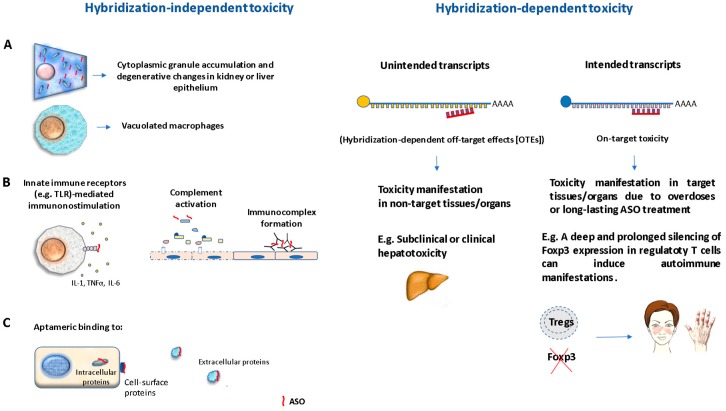
Types of ASOs-mediated toxicity: (1) Hybridization-independent toxicity represent those effects that are not due to Watson–Crick base pairing between an ASO and RNA. This type of toxicity occurs by three possible mechanisms: (**A**) ASOs accumulation effect is manifested as cytoplasmic granule accumulation, degenerative changes in kidney or liver epithelium, and presence of vacuolated macrophages. (**B**) Proinflammatory mechanisms due to ASOs interaction with innate immune receptors, inducing macrophages activation, complement activation, and immunocomplex formation. (**C**) Aptameric binding to intracellular cell surface or extracellular proteins. (2) Hybridization-dependent toxicity can be caused by partial or complete ASO interaction with unintended transcripts (hybridization-dependent off-target effects [OTEs]); or with intended transcripts (on-target toxicity).

The toxicity of ASOs is classified into two categories: hybridization-independent (non-pharmacologic) or hybridization-dependent (pharmacologically based) mechanisms [72] (Figure 2). Hybridization-independent toxicity is related to the specific chemical modification of the ASO and it does not involve base-pairing interactions. There are three general subcategories of hybridization-independent toxicities: (a) ASO effects by excessive accumulation, which is manifested by basophilic cytoplasmic granule accumulation, predominantly in kidney or liver epithelium that can produce degenerative changes. Another common histologic finding is the presence of increased granular macrophages or increased vacuolated macrophages related to cellular activation and proinflammatory cytokine production [72]; (b) immunomodulation (proinflammatory mechanisms, caused by toll-like receptors (TLR) and other innate immune receptors mediated mechanisms, complement proteins activation, and immune complexes causing immune cell activation and inflammatory reactions [72,73,74]. The major contributor to the proinflammatory effect is the backbone chemistry, although base-pair sequence and base modifications can also contribute. Proinflammatory effects are often observed as lymphoid hyperplasia in lymph nodes and spleen, associated to PTO ASOs, in rodents and monkeys, while reversible glomerulonephritis and vasculitis after administration of some types of ASOs have also been reported in monkeys [72]; (c) ASO interactions with extracellular, cell-surface, and/or intracellular proteins with high affinity and specificity (named as aptameric binding) [72,74]. According to Frasier [72], accumulation-related effects are the most encountered changes in preclinical toxicity studies.

The hybridization-dependent toxicity can be caused by either (a) hybridization-dependent off-target effects (OTEs) due to complete or partial complementary recognition of unintended transcripts or (b) hybridization-dependent toxicity (on-target toxicity). OTEs have been detected in preclinical studies after systemic administration of gapmers using biochemical markers of hepatotoxicity in the blood, while it has not been observed in clinical trials involving therapeutic ASOs to date [70]. Some authors also consider the non-pharmacologic effects as a form of off-target toxicity. On the other hand, on-target toxicity can occur after over-silencing intended transcripts or as a result of long-lasting treatment, and the adverse effects are specific to individual ASOs. For example, long lasting knockdown of genes involved in the immunosuppressive activity of regulatory T cells (Tregs) can potentially cause autoimmune manifestations.

Although the chemical modifications of ASOs have been important for their stability and delivery, the introduction of some structural modifications can increase the risk of toxicity. Swayze et al. [76] evaluated the toxicity of several ASOs containing either 2’-O-methoxyethylribose (MOE) or locked nucleic acid (LNA). They showed that incorporation of LNAs in some sequences induced hepatotoxic effects as early as 4 days after a single administration, whereas MOE-modified nucleotides in the same sequences did not cause toxicity. In another work, the authors observed that many unintended transcripts were downregulated in mice treated with hepatotoxic LNA ASOs, whereas in mice treated with non-hepatotoxic LNA ASOs, the transcript knockdown was highly selective [77].

Two forms of thrombocytopenia have been reported following several ASOs treatments. The most common form is mild, reversible, and dose-dependent thrombocytopenia, typically observed at high doses in monkeys and rodents after treatment with first-generation ASOs. In humans, transient thrombocytopenia without hemorrhagic manifestation has been reported after the use of first-generation ASOs such as oblimersen, aprinocarsen, ISIS 2503, and ISIS 5132 and less frequently with second-generation ASOs, including mipomersen, ISIS 104838, LY2275796 for cancer. A second form of thrombocytopenia with marked platelet depletion and hemorrhages is a rare but serious adverse event related to repeated exposure of ASOs [72,74,78].

### 2.4. Strategies to Improve ASOs Cell Targeting and Overcome Toxicity

Diverse reports have shown that naked ASOs are poorly internalized by cells, and they tend to be localized in endosomes, where they are unavailable for antisense purposes [47]. Thus, difficulties in biodistribution and cellular internalization are the main obstacles for clinical applications of ASOs. Diverse methods have been developed to improve cellular uptake of ASOs and their pharmacological activity, to increase their stability, to reduce the therapeutic dose, and to limit the off-target effects [79,80,81,82]. Conjugation of ASOs to molecules that can bind to certain ligands in the cell can improve their cell uptake and internalization. Small hydrophobic molecules, such as cholesterol [83], lipids [84], or fluorinated chains [85,86] may increase ASOs stability and their membrane permeation. Multivalent N-acetylgalactosamine (GalNAc) conjugation is another important way for ASOs delivery to hepatocytes [87]. Basic peptides such as *Drosophila melanogaster* homeotic transcription factor, *Antennapedia* peptide [88], and Tat protein of HIV-1 [89] have also been used to increase ASOs passage through the plasma membrane by a receptor- and transporter-independent mechanism delivering them directly into the cytoplasm and, hence, ultimately the nucleus.

In addition to direct conjugation of ASOs with defined molecules, the use of nanoparticles as vehicles for ASOs has been widely evaluated. The first generation of ASOs vehicles were liposomes, which are sphere-shaped vesicles consisting of one or more bilayers of phospholipids and cholesterol [90]. The ASO can be encapsulated into the aqueous compartment of the liposome or can be bound to the liposome surface by electrostatic interactions. Under physiological conditions, positively charged liposomes have high affinity for the negatively charged cell membranes and can easily bind to cells. Because these liposomes use the endosomal pathway to deliver ASOs into cells, they can be formulated with certain molecules inducing endosomal membrane destabilization, such as chloroquine and 1,2-dioleoyl-sn-glycero-3-phosphatidylethanolamine, to allow the scape of ASOs from the endosomes and be actively transported in high concentration to the nucleus [91,92,93,94,95].

Lipid nanoparticles (LNP) are other important formulations that have been used to enhance the delivery of ASOs to target tissues. Delivery using LNPs increases the stability and circulation time of ASOs [96,97]. LNPs contain ionizable amino lipids that self-assemble into nanoparticles when mixed with polyanionic oligonucleotides. The electrostatic interaction of LNPs with polyanionic nucleic acids promotes their encapsulation, allowing the escape to cell cytoplasm from the endosomal compartment [98]. Several ligands for overexpressed receptors on the target cell surface can be linked to LNPs, to facilitate the cellular uptake. These ligands include cell transferrin [99], penetrating peptides [100], folate [101], polysaccharides [102] and antibodies [103].

Besides liposomes and LNPs, cationic polymers, including poly-L-lysine [104,105], polyalkylcyanoacrylate nanoparticles [106,107,108]; polyethyleneimine (PEI) [109,110], and poly(amido amide) (PAMAM) dendrimers [111] have been also developed for ASOs delivery. These polyamines cause endosomal rupture via a “molecular sponge” mechanism and are less used than the cationic liposomes due to their toxicity [47]. Moreover, niosomes are an interesting alternative to liposomes for ASOs delivery. They are vesicles composed of non-ionic surfactants, amphipathic compounds with an overall neutral charge [80,112,113].

## 3. ASOs in Vaccines

The use of ASOs for vaccine improvement has been mainly based on the following strategies: (1) antigen modification; (2) targeting the host immune system by overexpression/inhibition of molecules involved in the immune response. In the following sections, we will analyze the main advances in these areas and the challenges still to be solved.

### 3.1. Antigen Modification

The first attempts using ASOs for antigen manipulation started in 1990. Goudsmit´s group used a phosphate-methylated ASO complementary to the tat responsive region (TAR) of the HIV-1 isolate CBL-4 (RUT) to reduce the viral infectivity [114,115]. However, some technical errors and interpretation of results that were subsequently corrected by the same authors caused the retraction of the article published in Science [116], as well as the conclusion issued that the observed inhibitory effect of viral infectivity should be ascribed to the phosphate methylation of natural DNA.

Tumor cells escape from immune surveillance by means of mechanisms to prevent tumor antigens recognition by the immune system. Several methods have been developed to increase the immunogenicity of the tumor cells. The most efficient methods can force tumor cells to present their own tumor antigens to the immune system [117]. In the early 1990s, the group led by Dr. Ostrand–Rosenberg demonstrated that tumor cells transfected with MHC class II molecules can generate a potent tumor cell vaccine, which protects against challenge with the parental tumor [118]. Moreover, supra-transfecting MHC class II+ tumor cells with *li* gene, coding for li protein (CD74), the invariant chain that normally blocks the binding of self-peptide fragments to MHC class II molecules, abrogated the immunogenicity of the modified cells [119].

**Figure 3:**
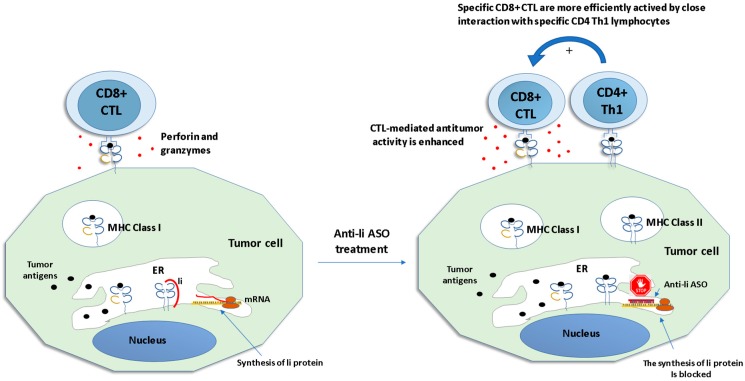
Tumor cells are forced to present their own tumor antigens to the immune system by anti-li ASO treatment. Left, MHC class I presents endogenous tumor antigens to CD8+ cytotoxic T cells (CTL). Ii protein blocks the binding of endogenous antigens to MHC class II in the endoplasmic reticulum (ER). Right, anti li-ASO blocks Ii protein expression, and endogenous tumor antigens are also presented by MHC class II molecules and recognized by specific Th1 lymphocytes. The simultaneous presentation of tumor antigens by both MHC class I to CTL and MHC II to Th1 lymphocytes induces a stronger antitumor response. (Adapted from [117]).

Based on this principle, Qiu et al. treated cancer cells expressing, naturally or by induction, MHC class II molecules and Ii protein, with anti-Ii ASO to induce the MHC-II–mediated presentation of diverse antigenic peptides to helper T cells (Figure 3). In each line of transfected tumor cells, the ASO profoundly suppressed Ii protein in 35% ± 55% cells, without affecting the expression of MHC class II molecules. The absence of the Ii protein increased the range of cancer-related epitopes presented to CD4+ helper T cells and generated effective tumor cell vaccines [120]. They also created several antisense Ii-reverse gene constructs (Ii-RGC) that inhibited Ii expression in A20 B lymphoma cells in vitro and Renca renal adenocarcinoma tumors in vivo. Subcutaneous Renca tumors in BALB/c mice were treated by intratumoral injection with a plasmid containing the gene for MHC class II transactivator (CIITA) and Ii-RGC. A subtherapeutic dose of IL-2 was also used to upregulate the activation of T cells. Significant tumor reduction and a decrease in the progression rates of the established tumors in the groups injected with Ii-RGC were observed, compared to the groups treated with IL-2 plus empty plasmid controls (*p* < 0.002) [121]. In another study, a single recombinant adenovirus with both interferon-gamma (IFN-γ) and Ii-RGC (rAV/IFN-γ/Ii-RGC) genes efficiently induced the MHC Class II+/Ii- phenotype in MC-38 colon adenocarcinoma cells and Renca tumors. Injection of tumor nodules with rAV/Ii-RGC and rAV/CIITA/IFN-γ, associated with a suboptimal dose of rAV/IL-2 induced a potent antitumor immune response. Control mice developed growing tumors by day 8 after injection. On the other hand, mice treated with rAV/CIITA/IFN-γ + rAV/IL-2 + rAV(wild type) showed delayed tumor growth in three of five mice, with tumor re-growing in two of these mice, resulting in one of five mice being tumor-free on day 60. Mice treated with rAV/CIITA/IFN-γ + rAV/IL-2 + rAV/Ii-RGC showed tumor regression in three of four animals. Finally, tumor-free mice were challenged on day 63 with Renca cells. Naive mice injected with the same number of Renca cells developed tumors while those tumor-free mice did not develop tumors in a follow-up of 34 days post-challenge. Similar results were observed in repeated experiments under the same conditions [122].

Rubenstein et al. evaluated the effect of bispecific ASOs targeting BCL-2 and epidermal growth factor receptor (EGFR) in the in vitro growth and prostatic antigen expression on androgen-sensitive human prostate adenocarcinoma (LNCaP) cells. Cultured cells were treated with 6.25 µM of either mono or bispecific ASOs and significant inhibition of the cellular growth was observed after treatment with bispecific ASOs. Interestingly, the bispecific ASO treatment also enhanced the expression of non-targeted proteins: prostate-specific cell surface antigens (PSMA), and IFN-γ. However, monospecific ASOs directed solely against BCL-2 did not stimulate the production of these proteins. The authors concluded that enhanced expression of cell surface differentiation antigens (such as PSMA) could increase their recognition and targeting by antitumor immunologic mechanisms and increase the effectiveness of tumor vaccines [123]. In other studies, the authors showed that LNCaP cells treated with ASOs directed against BCL2 administered in a nanoparticle suspension of lipofectin as vehicle exhibited non-target effects by suppressing the expression of apoptosis promoter caspase-3 [124]. In addition, they observed compensatory enhanced expression of other molecules such as (a) apoptosis inhibitor serine/threonine protein kinase (AKT1) [125], (b) androgen receptor (AR) and their co-activators p300 [126,127], (c) interleukin-6 (IL6) [128], (d) programmed death 1 (PD1) and its ligand PDL1, and (e) FAS-ligand, which activate apoptosis [129]. These and other reports of this group suggest that the use of ASOs to suppress BCL2 to restore apoptosis can lead to altered expression of non-targeted genes with different effects, including the stimulation of tumor proliferation [130].

### 3.2. Targeting Host Immune Mechanisms

It has been demonstrated that different isoforms of transforming growth factor-beta (TGF-β) with immunosuppressive activity are overexpressed in different malignant tumors such as melanoma, gliomas, prostate, gastric, colorectal, ovarian, gastric, and non-small cell lung cancers (NSCLC). Enhanced TGF-β2 expression in malignant cells is suggested to be a pivotal factor for tumor progression by inducing immunosuppression, metastasis, angiogenesis, and proliferation [131,132,133,134,135,136,137]. Tumor-infiltrating tolerogenic DCs and suppressor T cells are related to tumor-associated immunosuppression and tumor escape. These processes are mediated by TGF-β and IL-10 expression [133]. Elevated levels of TGF-β are inversely correlated with prognosis in patients with NSCLC [135,137].

The immunosuppressive effect of a TGF-β-producing autologous tumor vaccine was abrogated and rendered immunogenic when suppressing its TGF-β secretion with antisense strategy [138]. In that study, Tzai et al. used an MBT-2 tumor cell line [MBT-2/TGF-beta(-)#3] treated with ASOs against TGF-β and demonstrated that the amounts of this protein were significantly decreased in both irradiated and non-irradiated MBT-2/TGF-beta(-)#3 after 48 h of in vitro culture. This was associated to an increased expression of MHC class I molecule and Fas on the surface of MBT-2 tumor cells. This tumoral transformation enhanced vaccine immunogenicity and promoted a better survival rate in vaccinated mice when they were challenged with a two-fold higher amount of wild-type MBT-2 tumor cells.

Using a “double-punch” approach to overcome the escape of glioblastoma cells to the immune surveillance, [139] blocked the TGF-β production by TGF-β ASO. They used polybutyl cyanoacrylate nanoparticles (NPs) as vehicle for delivery of TGF-β ASO (NP-anti-TGF-β), to increase the immune response induced by active specific immunization with tumor cells infected with Newcastle-Disease-Virus (NDV). Glioblastoma cells were implanted into the brain of Fischer rats and then received intracutaneous vaccination with 1 × 10^5^ F98 cells infected with NDV. In addition, the rats were intraperitoneally injected with 9.34 nmol of TGF-β2 ASOs attached to 2.5 mg NPs coated with Polysorbate 80, suspended in sodium chloride solution. This treatment was repeated on days 1, 2, 10, 11, and 12 after tumor implantation. Three control groups were also used: one group was not treated at all, another group was treated by immunization only at days 0 and 10 and the third group only received ASOs attached to NPs without immunization. The treatment with NP-anti-TGF-β after immunization led to a rat mean survival rate of 25 days, which was significantly longer than the control animals’ survival. Moreover, the enhanced rat survival rate induced by the combined treatment was associated with reduced levels of TGF-β and increased rates of activated CD25+ T cells with significant differences to the control groups.

Belagenpumatucel-L (LucanixR), an allogeneic tumor cell vaccine gene-modified with TGF-β antisense, has been evaluated in locally advanced and metastatic NSCLC patients with an unfavorable response to chemotherapy. Results from a phase 2 trial showed a clear dose-dependent increase in overall survival (OS) with no significant adverse events [140]. A phase III trial that enrolled 270 patients treated with belagenpumatucel-L confirmed that the treatment was well tolerated. In contrast, there was no difference in survival between patients receiving belagenpumatucel-L compared with the placebo group, and there were no differences in progression-free survival [141].

Trabedersen [AP12009; OT-101] is a synthetic ASO that hybridizes with RNA sequences to block TGF-β translation, which is being used against advanced tumors overproducing TGF-β2 [142,143]. It has been reported that Trabedersen reduces the levels of this cytokine in human pancreatic cancer cell lines [136,142]. During a phase I/II clinical trial, Trabedersen improved OS in a subset of patients with advanced pancreatic cancer who received ASO treatment followed by subsequent chemotherapy. Levels of IL-8 and IL-15 were positively associated with OS across 12 of these patients and have been suggested as potential predictive biomarkers for this associated therapy in pancreatic cancer [144]. Trabedersen was also tested on patients with glioblastoma and anaplastic astrocytoma [145]. The ASO treatment exhibited an improved profile of efficacy and safety compared to that of conventional chemotherapy. More recently, it was reported that targeting TGF-β expression with two new ASOs named ISTH1047 and ISTH0047 results in strong anti-glioma activity in vitro and in vivo [146].

Another study demonstrated that immunization with C4HD, a hormone-dependent ductal breast tumor cell line, pretreated with PTO ASO against type I insulin-like growth factor receptor and irradiated, provided protection against C4HD wild-type tumor challenge. The ASO treatment induced expression of CD86 and heat shock protein 70 in the tumor cells. These molecules are involved in the induction of the immunogenic phenotype. Immunized mice exhibited a tumor growth inhibition of 53.4%, 61.6%, and 60.2% when compared with PBS-treated mice, wild-type C4HD cell-injected mice, and PTO ASO-treated C4HD cell-injected mice, respectively. The specificity of the antitumor effect was proved since no cross-protection was observed against other syngeneic mammary tumor cell lines. In addition, immunization induced splenocytes to produce Ag-dependent IFN-γ, indicating the presence of an antitumor Th1 response. Moreover, a cellular CD8+-dependent immune response, acting through the Fas/Fas ligand death pathway, was observed [147].

Our group evaluated the effect of silencing Foxp3 on antitumor efficacy of a genetically modified tumor cell vaccine against B16 mouse melanoma cells. Miguel et al. transplanted B16 mouse tumor cells to mice prior to treating them with irradiated GM-CSF (granulocyte and macrophage colony-stimulating factor) tumor-producing cells combined with anti-Foxp3 2’-O-methyl phosphorotioate-modified oligonucleotides (2’-OMe-PS-ASOs). Antitumor response and mice survival rate improved in animals treated with therapeutic vaccine combined with Foxp3 antisense when compared to vehicle-treated control. In that study, an ASO against CTLA4 was also evaluated, but this resulted less efficacious than anti-Foxp3. These data supported the hypothesis that silencing Foxp3 can be a potential adjuvant strategy to improve antitumor vaccines based on the reduction of Treg-mediated immunosuppressive effects in the tumor microenvironment [148].

#### ASOs as Vaccine Adjuvants in Subunit Vaccines

**Figure 4:**
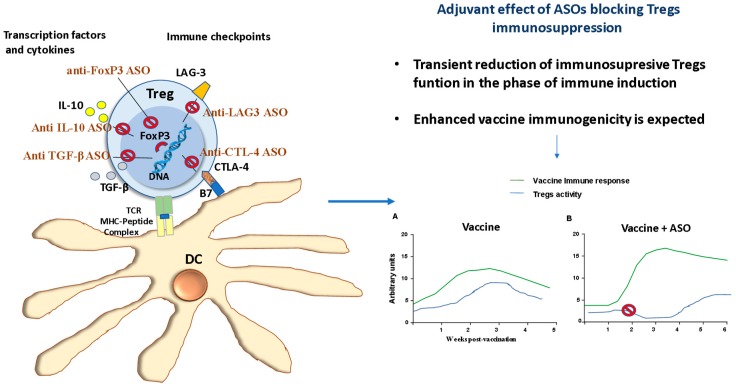
Some examples of the use of ASOs as vaccine adjuvant by modulating the regulatory T cells (Tregs) response. Left, molecules involved in Tregs function that are currently being studied as target for vaccine improvement with ASOs. CTLA-4, T-lymphocyte antigen4; DC, dendritic cells; IL-, interleukin; LAG-3, lymphocyte activation gene-3; TGFβ, transforming growth factor beta; TCR, T cells receptor. Right, (**A**) Normal post-vaccination immune response without Tregs modulation. (**B**) ASO-mediated transitory Tregs depletion/inhibition elicit a stronger vaccine immune response.

In the last years, there has been a growing interest in the rational design of vaccines using defined molecules with well-characterized cellular and molecular mechanisms of action. One of the current directions of this approach is the development of subunit vaccines that contain only the minimal microbial component necessary to stimulate long-lasting protective or therapeutic immune responses [149]. In the meantime, another direction is targeting immune regulatory networks with molecular adjuvants for improving vaccine immunogenicity with the lowest possible toxicity [150]. Several ASOs have been evaluated as adjuvants to enhance the immune response in experimental vaccines. These ASOs were designed against suppressor components such as cytokines [151,152], checkpoints [153,154], or transcription factors [148] (Figure 4).

Using a neonatal mouse model of respiratory syncytial virus (RSV) infection, Ripple et al. evaluated if local inhibition of IL-4Rα expression using an ASO specific for IL-4Rα during primary RSV infection would prevent Th2-biased responses to secondary RSV infection and improve long-term pulmonary function. Mice were initially infected with RSV at one week after birth and re-infected at six weeks of age. Intranasal administration of IL-4Rα ASO during primary RSV infection does not hinder viral clearance; however, the ASO treatment abolished the pulmonary dysfunction normally observed following reinfection in the adult with a significant response (*p* < 0.05) compared with non-treated mice. The parameters evaluated were lung resistance in response to increasing doses of methacholine (MeCh) and histology after secondary RSV infection, including a measure of % inflammation and mucus index. This protection was achieved by decreasing the Th2 immune modulation responses associated with an increased Th1 immune activation (i.e., elevated Th1 cell numbers and type I antibodies and cytokines). The authors suggested that vaccine strategies based on IL-4Rα ASO might offer significant benefits to preventing RSV-mediated pulmonary disease in infants [151].

In a recent report, Zhang et al. [152] evaluated the effect of an interleukin 10 (IL-10)- PTO targeted ASO as an immune adjuvant in intradermal vaccination using ovalbumin (OVA), a standard T-dependent antigen. Their results showed that the specific antibody titer of OVA increased 100-fold upon the addition of IL-10 ASO as adjuvant compared to that of OVA alone (*p* < 0.01). According to the authors, IL-10 ASO potentiated the immune response in a similar way to that of Freund’s incomplete adjuvant, used as the positive control, without detectable cell or tissue toxicity. They also confirmed that IL-10 ASO enhanced the T-mediated specific immune responses by temporal inhibition of the IL-10 produced by local DCs.

The synergistic effect of two ASOs against cytotoxic T lymphocyte antigen 4 (CTLA-4), a widely studied checkpoint inhibitor of T-cell proliferation and activation, was evaluated in experimental vaccines. These vaccines were prepared with either recombinant PCV2b capsid protein or inactivated foot-and-mouth disease virus (FMDV) in ICR and BALB/c mice. The sequences of these anti-CTLA-4 ASOs, named CMD-1 and CMD-2, were complementary to conserved regions that are identical between human and mouse CTLA-4 mRNA present in 3’ untranslated region (3’ UTR) [153]. The authors found that CMD-1 inhibited the antigen-induced CTLA-4 up-regulation on the CD4+ T cells and enhanced the antibody response against both recombinant PCV2b capsid protein and inactivated FMDV in both ICR and BALB/c mice compared with the control group without ASOs (*p* < 0.05). Moreover, CMD-1 promoted high expression levels of CD80 and CD86 on the CD11c+ populations and the recalled proliferation of CD4+ T cells and CD19+ B cells.

In another recent study, the same group designed an interfering ASO (LIO-1) against lymphocyte activation gene-3 (LAG3) to enhance the immune response induced by both ISA35- formulated recombinant protein vaccines and ISA35-formulated inactivated influenza virus vaccines. LAG3 is a transmembrane protein expressed on activated T cells that triggers inhibitory signals for the activation of B cells to produce antibodies. The authors demonstrated that LIO-1 induced the degradation of LAG3 mRNA and decreased the LAG3 expression on CD4+ T cells, promoting the activation and increasing the production of IFN-γ, IL-2, and IL-6 CD4+ T cells re-stimulated with specific antigens. Moreover, they found that LIO-1 enhanced the antibody responses induced by both vaccine formulations in mice [154].

## 4. Challenges and Opportunities for ASOs Application in Vaccinology

Since the discovery, more than two decades ago, that ASOs could be used in clinical pharmacology to modulate protein expression, several antisense drugs have been approved for clinical use in the last years. Nowadays, there is a great interest in ASOs-based drugs, due to the development of more specific and nuclease resistant structures, as well as more efficient vehicles that enhance the ASOs delivery to target tissues. The application of ASOs to improve vaccines is more recent but undoubtedly with promissory perspectives. They can be used for antigen modification of whole cell immunogens or as vaccine adjuvant by enhancing the host immune response.

Although most of the uses of ASOs in vaccines have been directed to the transformation of tumor cells to increase their immunogenicity [117,155], various factors including the tumor microenvironment complex [156], constitute real challenges to achieve homogeneous results.

Only recently it has been reported that it is possible to successfully use ASOs as part of vaccine formulation for single antigens [151,152,153,154]. This strategy can avoid the administration of systemic higher doses of ASOs, potentially reducing undesired events such as off-target effects and adjuvant-mediated immunotoxicity [157,158]. However, despite their specificity and broadness of use, some problems remain unsolved in ASOs for vaccine use. The experiences in the use of ASOs as adjuvants to improve the immune response are still scarce, and the possibility of toxicity by immune overstimulation needs to be deeply studied. ASO toxicities including off-target effects can be both sequences- and chemistry-dependent, and thus, each ASO molecule must be considered independently during toxicological studies [159]. In this way, bioinformatic tools are being developed to identify suitable target regions and to analyze potential off-target effects of therapeutic ASOs [157]. A recent guideline offers a set of recommendations and standards for designing and evaluating experiments using ASOs and double-stranded RNAs that help to achieve a better interpretation of data in the pharmacological evaluation of these molecules [69]. This list summarizes several of the current trends in ASOs research for vaccine development:

- Discovery of new suitable genes to improve vaccine protective immunogenicity against specific infectious or tumoral disease using ASOs.
- Development of bioinformatic tools and in vitro systems for ASOs screening to vaccine application.
- Discovery of delivery systems that can promote effective ASOs cellular uptake in the immune system.
- Studies of stability and antigen-ASOs compatibility in vaccine formulations.
- Immunotoxicity studies to discover potential consequences of immune overstimulation.
- Studies of efficacy/safety in different genetic contexts.

## 5. Concluding Remarks

Roughly 40 years have passed since the birth of synthetic ASOs, meanwhile the medical application of ASOs has advanced rapidly in understanding and clinical/regulatory acceptance. Herein, we have reviewed recent progress in ASOs research focusing on prophylactic and therapeutic vaccine applications. The widespread availability of various types of ASOs with well-characterized structures and mechanisms of action suggests that this is an emerging field of potential application for the next generation of vaccines. Experimental and clinical evidence shows that ASOs can be used to control the expression of certain genes, favoring the induction of stronger antigen immune responses. Recent reports suggest that ASOs can be use as vaccine adjuvant, but further studies are necessary to provide a better understanding of the ASOs-mediated immunostimulation and potential risk of toxicity. The next few years promise relevant achievements in this emergent area.

## Figures and Tables

**Table 1:** Summary of three generations of the most studied ASOs chemical modifications.

Chemical Modifications	Characteristics	Mechanisms	Clinical Use	Limitations
First Generation				
Phosphorothioate (PTO), Methylphosphonate (MPO)	Either a sulfur atom (PTO), or a methyl group (MPO) substitutes the non-bridging oxygen atoms in the phosphodiester bond.	First generation ASOs promote degradation of target mRNA by RNase H enzyme. They also confer higher solubility, resistance to nuclease degradation, antisense activity and longer plasma half-life as compared with phosphodiester oligonucleotides.	PTO is the most widely used modification of ASOs. Fomivirsen, is a PTO-modified ASO, used as local treatment of cytomegalovirus (CMV) retinitis in patients with acquired immunodeficiency syndrome (AIDS) [48].	High affinity for various cellular proteins and components of the innate immune system, such as Toll-like receptors (TLRs), with proinflammatory effects. Commonly reported side effects following systemic administration of PTO ASOs include fever, activated partial thromboplastin time prolongation, thrombocytopenia, and leukopenia.
**Second Generation**				
ASOs with 2’-O-alkyl modifications of the ribose. Chimeric ‘gapmer’ ASOs	2’-O-Methyl (2’-OMe) and 2’-O-Methoxyethyl (2’-MOE) are the most widely studied. Chimeric ‘gapmer’ ASOs consist in a central ‘gap’ region containing 10 DNA or PTO DNA monomers, flanked on both 5’ and 3’extremities by alkyl modified nucleotides such as 2′-OM or 2’-MOE.	The PTO DNA induces RNase H cleavage while 2′-OME or 2′-MOE on both sides (5′- and 3′-directions) confers nuclease-resistance, and they can exert activity by a steric interference of translation process. They are safer than PTO-modified ASOs and exhibit enhanced affinity towards the complementary RNA with better tissue uptake and longer in vivo half-life.	Mipomersen is used as an adjunct therapy for homozygous familial hypercholesterolemia [49]. Nusinersen was approved for spinal muscular atrophy treatment [50]. Apatorsen is a HSP27 targeting ASO that is being studied in phase II clinical trials in patients with metastatic castration resistant prostate cancer [51] and Untreated Stage IV Non-Squamous-Non-Small-Cell Lung Cancer [52].	A subset of 2´-MOE-modified ASOs induced pro-inflammatory cytokines and type I interferons (IFN-α/β) and interaction with innate immune receptors such as TLR9, melanoma-differentiation associated-5 (MDA-5) and IFN-β promoter stimulator-1 (IPS-1).
**Third Generation**				
Peptide nucleic acid (PNA)	PNA is a synthetic DNA in which the deoxyribose phosphate backbone is replaced by polyamide linkages.	PNA block the protein expression, by steric hindrance, forming sequence-specific duplex with the targeted mRNA. They are biologically stable and have good hybridization properties.	The potential of PNA as drugs in gene therapy has been hampered by the poor intrinsic uptake of PNA by living cells. Current strategies for improving PNA delivery into the cytosolic space and nucleus include microinjection, electroporation, co-transfection with DNA, or conjugation to lipophilic moieties, nanoparticles, cell-penetrating peptides (CPPs), oligo-aspartic acid, or nuclear localization signal (NLS) peptides to enhance cellular internalization	PNA do not activate the RNase H to cleave the target hybridized RNA. PNA have low solubility and cellular uptake.
Phosphoramidate morpholino oligomer (PMO)	PMOs are neutral ASOs. The pentose sugar is substituted by a morpholino ring and the inter-nucleotide linkages are phosphoramidate bonds in place of phosphodiester bonds.	The mechanism of PMO is the translational arrest mediated by steric interference of ribosomal assembly. PMO show fewer nonspecific properties and lesser toxicity than PTO.	Eteplirsen was approved for Duchenne muscular dystrophy (DMD) treatment [53]. Other potential applications include the treatment of viral infections, antibiotic-resistant bacterial infections, and cancers [54].	PMOs exhibit reduced cellular uptake. Conjugation with peptides such as arginine-rich peptide (ARP) can enhance its cellular uptake and antisense efficacy.
Locked nucleic acid (LNA)	LNAs are chemically modified nucleotides with a ribose containing a methylene bridge between the 2′-oxygen and the 4′-carbon of the ribose.	LNA modifications improve the affinity of ASO hybridization towards mRNA target, by increase of the DNA/RNA heteroduplexes thermal stability. LNAs avoid nuclease degradation.	Diverse LNAs are currently in clinical trials by several biotechnology firms.	LNA does not activate RNase. LNA nucleotides can be incorporated at the ends of RNA and DNA sequences to form chimeric oligonucleotides resulting in restoration of RNase H-mediated cleavage of mRNA.

**Table 2:** Key advantages of ASOs compared to monoclonal antibodies.

	ASOs	mAb
**Molecular Weight**	~6 to10 kDa	~150 kDa
**Structure**	Relatively simple structure. Usually 13–20 mer with chemical modifications	Glycoproteins with complex structure
**Thermal Stability**	Highly stable. Lyophilization and freezing does not modify its biological activity	Low stability. Cold chain through the storage, handling, and transportation is necessary
**Cellular Bioavailability**	ASOs can penetrate the cells and act on intracellular targets	They are unable to penetrate the cells
**Immunogenicity**	ASOs are not properly immunogenic	Highly immunogenic by xenogeneic differences, e.g., between mice and humans
**Specificity**	Highly specific but off target interaction can be observed	Highly specific but cross-reactivity can be observed
**Toxicity**	Relatively low toxicity	Different grades of toxicity have been described
**Development and Manufacturing**	ASOs are obtained synthetically.The use of vehicles can add complexity to the manufacture process	The production for pharmacological proposes requires high level of technological complexity
